# Effectiveness of Cardiac Rehabilitation in Enhancing Adherence and Improving Clinical Outcomes Post‐Acute Coronary Syndrome: A Randomized Controlled Trial

**DOI:** 10.1002/clc.70160

**Published:** 2025-06-02

**Authors:** Haşim Tüner, Fuat Polat, Enes Alıç, Ali Nail Kaya, Çiğdem Bahar Çakmak, Ferhat Coşkun, Emrah Özbek

**Affiliations:** ^1^ Department of Cardiology Istanbul Arel University Memorial Bahcelievler Hospital İstanbul Türkiye; ^2^ Department of Cardiology Dr. Siyami Ersek Thoracic and Cardiovascular Surgery Educatıon Research Hospıtal İstanbul Türkiye; ^3^ Health Sciences University Istanbul Turkey; ^4^ Department of Cardiology Istanbul Aydin University VM Medical Park Florya Hospital İstanbul Türkiye; ^5^ Department of Cardiology Hakkari State Hospital Hakkari Türkiye; ^6^ Department of Cardiology Samsun Gazi State Hospital Samsun Türkiye; ^7^ Department of Cardiology Batman Health Sciences University Training and Research Hospital Batman Türkiye; ^8^ Department of Cardiology Van Health Sciences University Training and Research Hospital Van Türkiye

**Keywords:** acute coronary syndrome, adherence, cardiac rehabilitation, clinical outcomes

## Abstract

**Background:**

Acute coronary syndrome (ACS) remains a major contributor to cardiovascular morbidity and mortality. Cardiac rehabilitation programs have shown promise in improving adherence to lifestyle and medical recommendations, yet their impact on clinical outcomes and complications requires further investigation.

**Methods:**

This prospective, randomized, single‐center study evaluated the effects of cardiac rehabilitation on adherence and clinical outcomes in ACS patients. A total of 340 patients were randomized into a Cardiac Rehabilitation Group or Control Group. The Cardiac Rehabilitation Group underwent supervised exercise, dietary counseling, and education, while the Control Group received standard recommendations. Outcomes, including adherence rates and complications, were assessed over 1 year, with additional interim analyses to evaluate early sustainability of behavioral changes.

**Results:**

Patients in the Cardiac Rehabilitation Group demonstrated significant improvements in adherence to dietary recommendations (73.5% vs. 52.4%, *p* < 0.01) and physical activity (85.3% vs. 68.2%, *p* < 0.01). Cardiac Rehabilitation Group patients also experienced fewer instances of weight gain (22.9% vs. 34.7%, *p* = 0.017) and access site complications (21.2% vs. 40%, *p* < 0.01). Hospital readmissions were reduced in the Cardiac Rehabilitation Group compared to the Control Group (18.8% vs. 31.2%, *p* = 0.015). Non‐adherence to dietary recommendations (HR: 2.42, 95% CI: 1.08–5.41, *p* = 0.032) and medical treatments (HR: 2.84, 95% CI: 1.32–6.11, *p* = 0.007) were significantly associated with increased risk of revascularization.

**Conclusion:**

Cardiac rehabilitation significantly enhances adherence to medical and lifestyle recommendations, reduces complications, and improves outcomes in ACS patients. These findings emphasize the critical role of structured rehabilitation in post‐ACS management.

## Summary

- Cardiac rehabilitation significantly enhanced adherence to diet and exercise after ACS.
- Participation in rehabilitation reduced the incidence of weight gain and access site complications.
- Poor adherence to rehabilitation was linked to increased revascularization within 1 year.

## Graphical abstract

Cardiac rehabilitation significantly improved dietary adherence, physical activity, clinical outcomes, and psychological well‐being in acute coronary syndrome patients over 1 year, reducing revascularization rates and hospital readmissions. Non‐adherence to treatment and diet were key predictors of poor outcomes.

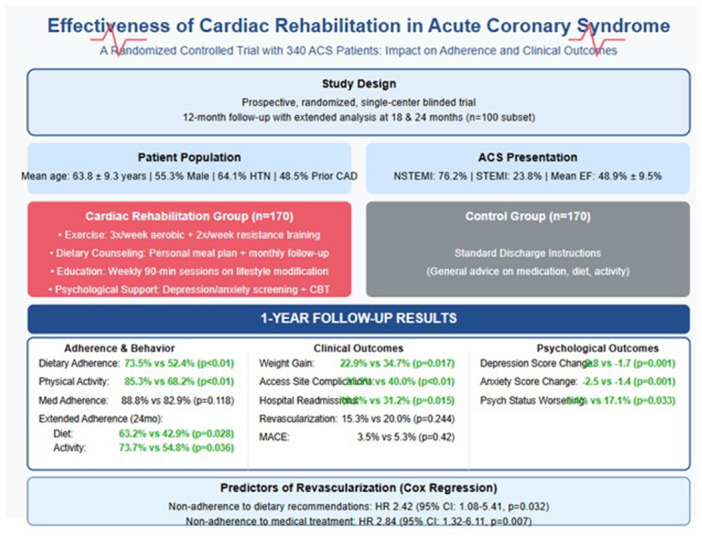

## 1 Introduction

Acute coronary syndrome (ACS), encompassing conditions such as ST‐elevation myocardial infarction (STEMI), non‐STEMI (NSTEMI), and unstable angina, is a significant contributor to cardiovascular morbidity and mortality worldwide [1]. Despite advancements in pharmacological treatments, revascularization techniques, and preventive strategies, patients recovering from ACS remain at high risk for recurrent cardiovascular events, hospitalizations, and adverse outcomes [2]. Effective secondary prevention strategies are critical for mitigating this risk and improving long‐term health outcomes [3].

Secondary prevention involves a combination of pharmacological therapy and non‐pharmacological interventions such as lifestyle modification, risk factor control, and behavioral changes [4, 5, 6]. Key elements include adherence to prescribed medications, maintaining a heart‐healthy diet, engaging in regular physical activity, smoking cessation, and weight management. However, achieving sustained adherence to these recommendations remains challenging in real‐world clinical practice [7, 8]. Non‐adherence has been consistently linked to worse clinical outcomes, including increased risk of rehospitalization, revascularization, and mortality [9].

Cardiac rehabilitation (CR) programs are a cornerstone of secondary prevention in cardiovascular disease. These multidisciplinary interventions typically include supervised exercise training, dietary counseling, psychosocial support, and education aimed at empowering patients to actively manage their condition [10, 11]. Studies have shown that CR can improve functional capacity, quality of life, and adherence to lifestyle and medical recommendations [4, 11, 12]. Despite these benefits, participation in CR programs is suboptimal, with many patients either not referred to or not completing these programs [5, 13, 14]. Additionally, while the overall benefits of CR are well‐documented, there is a need for more detailed evidence on its specific effects on adherence behaviors, clinical outcomes, and complications in patients with ACS.

This study aims to address this gap by evaluating the impact of CR training on adherence to medical and lifestyle recommendations, clinical outcomes, and the incidence of complications in a cohort of patients recovering from ACS. Furthermore, the study seeks to identify factors associated with the need for revascularization within 1 year, providing insights into the predictors of adverse outcomes. By highlighting the role of structured rehabilitation programs in post‐ACS care, this study aims to support their broader implementation as a strategy to improve patient outcomes and reduce the burden of cardiovascular disease.

## 2 Methods

### 2.1 Study Design and Population

This was a prospective, randomized, single‐center single‐blind study conducted to investigate the impact of CR training on adherence to medical and lifestyle recommendations, clinical outcomes, and complications in patients recovering from ACS. To mitigate the risk of detection bias, outcome assessors were blinded to group allocation through the following measures:

- A separate team of research personnel, who were not involved in patient treatment or group allocation, was responsible for collecting and evaluating all study outcomes.
- Outcome measurement tools and procedures were predetermined and uniformly applied across both study groups to minimize potential bias.
- All patient identifiers related to group allocation were removed from data collection forms before analysis.

The study also aimed to identify predictors of revascularization within 1 year post‐ACS. Patients were those admitted to a secondary care hospital with ACS between July 2021 and February 2023.

The inclusion criteria were:

1. Age ≥ 18 years.
2. Confirmed diagnosis of ACS (STEMI, NSTEMI, or unstable angina) based on clinical, electrocardiographic, and biomarker findings.
3. Hemodynamic stability at discharge.
4. Willingness to participate in follow‐up visits for a duration of 1 year.

Exclusion criteria included:

1. Advanced heart failure (New York Heart Association Class IV).
2. Severe comorbidities such as advanced chronic kidney disease (eGFR < 30 mL/min/1.73 m²), end‐stage liver disease, or malignancy.
3. Significant musculoskeletal or neurological disorders preventing physical activity.
4. Cognitive impairment or inability to provide informed consent.

Eligible patients were randomized in a 1:1 ratio to either the CR Training Group or the Control Group using a computer‐generated randomization sequence. Allocation was concealed until patients were assigned to their respective groups.

### 2.2 CR Training Group

Patients in this group participated in a structured outpatient CR program supervised by a multidisciplinary team of cardiologists, physiotherapists, dietitians, and psychologists. The program was designed as a comprehensive 12‐week intervention with four key components, each meticulously tailored to address the complex needs of ACS patients.

#### 2.2.1 Exercise Training

The exercise training component began with a comprehensive initial assessment, including cardiopulmonary exercise testing and individualized risk stratification. Patients engaged in a structured exercise regimen consisting of supervised aerobic and resistance training. Aerobic exercises were conducted three times per week, with sessions lasting 30−45 min at a moderate intensity (60%−70% of peak heart rate). Exercises included treadmill walking, stationary cycling, and low‐impact activities, with progression carefully monitored and adjusted based on individual patient response. Resistance training was incorporated twice weekly, focusing on major muscle groups using resistance bands, light weights, and body‐weight exercises. Throughout the program, patients were monitored with continuous cardiac telemetry, regular assessments of perceived exertion, and periodic reevaluations of exercise tolerance.

#### 2.2.2 Dietary Counseling

Dietary counseling was equally comprehensive, beginning with an in‐depth nutritional assessment that included metabolic profiling and a detailed dietary habit questionnaire. Patients received individualized 60‐min consultations with a registered dietitian, resulting in personalized meal plans targeting specific nutritional goals. These goals included sodium reduction to less than 2300 mg per day, limiting saturated fat intake to less than 7% of total calories, increasing fiber intake to 25−30 g daily, and implementing portion control strategies. Follow‐up support included monthly individual counseling sessions, quarterly group nutrition workshops, and access to educational materials and a smartphone application for dietary tracking.

#### 2.2.3 Education

The educational component consisted of weekly 90‐min group sessions covering critical aspects of cardiac health management. Topics included medication management, risk factor modification, stress management, symptom recognition, and lifestyle strategies. Educational materials were diverse, including illustrated guidebooks, video‐based resources, and interactive digital modules. These sessions provided in‐depth information on the pathophysiology of ACS, the importance of medication adherence, and comprehensive cardiovascular risk reduction strategies.

#### 2.2.4 Psychological Support

Psychological support was integrated through a structured approach beginning with standardized screening for depression, anxiety, and stress. Patients were offered up to six individual counseling sessions, optional group support meetings, and training in cognitive‐behavioral stress management and mindfulness techniques. Quarterly follow‐up assessments ensured ongoing psychological support, with referrals to specialized mental health services when necessary.

The program concluded with a personalized maintenance plan, providing recommendations for continued independent exercise and 6 months of telephone support to ensure sustained lifestyle modifications. This holistic approach aimed to empower patients with the knowledge, skills, and support necessary for long‐term cardiovascular health management.

### 2.3 Control Group

Patients in the control group received standard discharge instructions from their attending physicians. These instructions included general advice on medication adherence, dietary modifications, physical activity, and smoking cessation but did not include structured or supervised sessions.

### 2.4 Baseline Data Collection

At enrollment, comprehensive baseline data were collected, including:

- *Demographic information:* Age, gender, and socioeconomic status.
- *Clinical characteristics:* Presence of comorbidities such as hypertension, diabetes mellitus, and prior coronary artery disease.
- *Laboratory parameters:* White blood cell count (WBC), hemoglobin levels, and estimated glomerular filtration rate (eGFR).
- *Echocardiographic findings:* Left ventricular ejection fraction (EF) and any significant structural abnormalities.
- *Hospitalization data:* Type of ACS (STEMI, NSTEMI, or unstable angina) and duration of hospital stay.

### 2.5 Follow‐Up and Outcome Measures

Blinded outcome assessment was implemented for the following measures:

Patients were followed for 1 year with structured clinic visits and telephone interviews at 1, 3, 6, 9, and 12 months, and a subset of participants (*n* = 100, randomly selected) underwent extended follow‐up at 18 and 24 months to assess long‐term adherence trends. Both the intervention and control groups were contacted and assessed at the same predefined intervals using identical follow‐up schedules. Clinic visits and telephone interviews were conducted by the same blinded outcome assessors across both groups to ensure consistency in data collection and to minimize bias. The control group did not receive structured rehabilitation sessions but was followed with the same rigor and frequency as the intervention group to ensure methodological comparability. Outcomes were categorized as primary (adherence measures) and secondary (clinical events/complications):

### 2.6 Primary Outcomes

- *Adherence to medical treatment:* Adherence was assessed using a multimodal approach to increase reliability. In addition to pharmacy refill records and pill counts during clinic visits, we administered the validated 8‐item Morisky Medication Adherence Scale (MMAS‐8), which has been widely used in cardiovascular research. A score of ≥6 on the MMAS‐8 was considered indicative of good adherence. These assessments were conducted by blinded research staff to minimize reporting bias.
- We evaluated adherence specifically to evidence‐based medications prescribed at discharge, including antiplatelet agents (aspirin, P2Y12 inhibitors), beta‐blockers, statins, and angiotensin‐converting enzyme (ACE) inhibitors or angiotensin receptor blockers (ARBs).
- *Adherence to dietary recommendations:* Assessed using the Mediterranean Diet Score (MDS; range 0–14), with adherence defined as MDS ≥ 10 by independent nutritional assessors unaware of group allocation.
- *Adherence to physical activity:* Measured via accelerometer data (≥150 min/week moderate activity) and self‐reported logs with data analysis performed by blinded investigators.

### 2.7 Secondary Outcomes

- *Revascularization:* Repeat PCI or CABG, adjudicated by an independent blinded committee.
- *Weight gain:* Increase ≥ 2 kg from baseline measured during clinic visits by blinded research staff.
- *Smoking cessation:* Biochemically verified (exhaled CO < 6 ppm) at 12 months by blinded assessors.
- *Access site complications:* Hematoma (>5 cm), infection (clinical + culture confirmation), or vascular injury requiring intervention, evaluated by blinded clinicians.
- *Hospital readmissions:* All‐cause and cardiovascular‐related, verified via electronic records by blinded research personnel.
- *Psychological outcomes:* Psychological status was assessed using two validated instruments administered by blinded mental health professionals:
  Depression: Measured using the Patient Health Questionnaire‐9 (PHQ‐9), a validated 9‐item instrument scored from 0 to 27, with scores ≥ 10 indicating moderate‐to‐severe depressive symptoms
  Anxiety: Assessed using the Generalized Anxiety Disorder‐7 (GAD‐7), a validated 7‐item instrument scored from 0 to 21, with scores ≥ 8 indicating moderate‐to‐severe anxiety symptoms
  Both instruments were administered at baseline and at 3, 6, and 12 months follow‐up—Clinically significant worsening was defined as an increase of ≥5‐points in either scale from baseline to 12‐month assessment, or development of moderate‐to‐severe symptoms in previously asymptomatic patients
- *Statistical power calculation:* Assuming 30% non‐adherence in controls and 15% in the intervention group (*α* = 0.05, *β* = 0.20), 170 patients per group provided 85% power.

### 2.8 Safety Monitoring and Adverse Events

Safety was a primary concern throughout the study, particularly during exercise sessions. We defined and monitored the following adverse events:

*Exercise‐related adverse events:* Any cardiovascular symptoms (chest pain, dyspnea disproportionate to exercise level, palpitations, dizziness, pre‐syncope), arrhythmias detected during telemetry monitoring, abnormal blood pressure responses (systolic blood pressure > 250 mmHg or diastolic blood pressure > 115 mmHg, or a decrease in systolic blood pressure > 10 mmHg during exertion), or musculoskeletal injuries occurring during or within 3 h after exercise sessions.

Dietary intervention‐related adverse events: Any adverse reactions to dietary recommendations, including gastrointestinal disturbances, allergic reactions, or significant unintended weight loss (>5% of body weight in 1 month).

*Major adverse cardiovascular events (MACE):* Death, myocardial infarction, hospitalization for unstable angina, stroke, or unplanned revascularization.

All exercise sessions were supervised by medical professionals trained in advanced cardiac life support. Each session began with symptom assessment and vital sign measurements. Exercise intensity was adjusted based on individual tolerance and clinical status. Emergency protocols and equipment were in place for immediate intervention if needed.

An independent safety monitoring board reviewed all adverse events monthly to determine relatedness to the intervention and recommend protocol modifications if necessary.

### 2.9 Statistical Analysis

Continuous variables were presented as mean ± standard deviation or median (interquartile range) depending on data distribution, which was assessed using the Shapiro−Wilk test. Categorical variables were expressed as frequencies and percentages.

Baseline characteristics and outcomes were compared between the two groups using:

- Independent *t*‐tests for normally distributed continuous variables.
- Mann−Whitney *U* tests for non‐normally distributed continuous variables.
- Chi‐square tests or Fisher's exact tests for categorical variables.

Univariate and multivariate Cox proportional hazards regression analyses were performed to identify factors associated with revascularization within 1 year. For adherence variables, “adherent” was set as the reference category (coded = 1) versus “non‐adherent” (coded = 0) to ensure hazard ratios (HR) > 1 would reflect increased risk associated with non‐adherence. Variables with a *p* < 0.1 in the univariate analysis were included in the multivariate model. Additionally, potential confounding variables including socioeconomic status indicators (education level, income bracket, employment status), baseline medication use (pre‐admission antiplatelet agents, statins, beta‐blockers, ACE inhibitors/ARBs), and psychosocial variables (baseline PHQ‐9 and GAD‐7 scores) were included in the multivariate model regardless of their univariate p‐values, as these factors may influence both adherence behaviors and clinical outcomes. Results were expressed as HR with 95% confidence intervals (CI).

A statistical power calculation was performed before recruitment to ensure adequate power to detect clinically meaningful differences, particularly in the primary behavioral outcomes and secondary outcomes such as revascularization. Based on prior literature and clinical expectations, we assumed a 30% non‐adherence rate to medical or lifestyle recommendations in the control group and a 15% rate in the intervention group. To detect this 15% absolute difference with 80% power and a two‐sided alpha level of 0.05, a minimum of 153 patients per group was required. Allowing for an anticipated 10% dropout rate, the final sample size was increased to 170 patients per group (total *N* = 340). This sample size also provides sufficient power to detect a HR of approximately 0.40 in revascularization rates between groups, assuming an event rate of 15%–20% at 1 year.

All statistical analyses were performed using SPSS software (version 25.0, IBM Corp., Armonk, NY, USA). A two‐sided *p* < 0.05 was considered statistically significant.

### 2.10 Ethical Considerations

The study was conducted in accordance with the Declaration of Helsinki and was approved by the institutional ethics committee with the date 29.07.2021 and approval number 2021/15. Written informed consent was obtained from all participants before enrollment. Patient confidentiality and data integrity were maintained throughout the study.

## 3 Results

A total of 340 patients with ACS who were followed up at the same center were included in the study. Half of the patients were randomly assigned to participate in CR training, while the other half received only routine physician recommendations without additional training.

**Figure 1:**
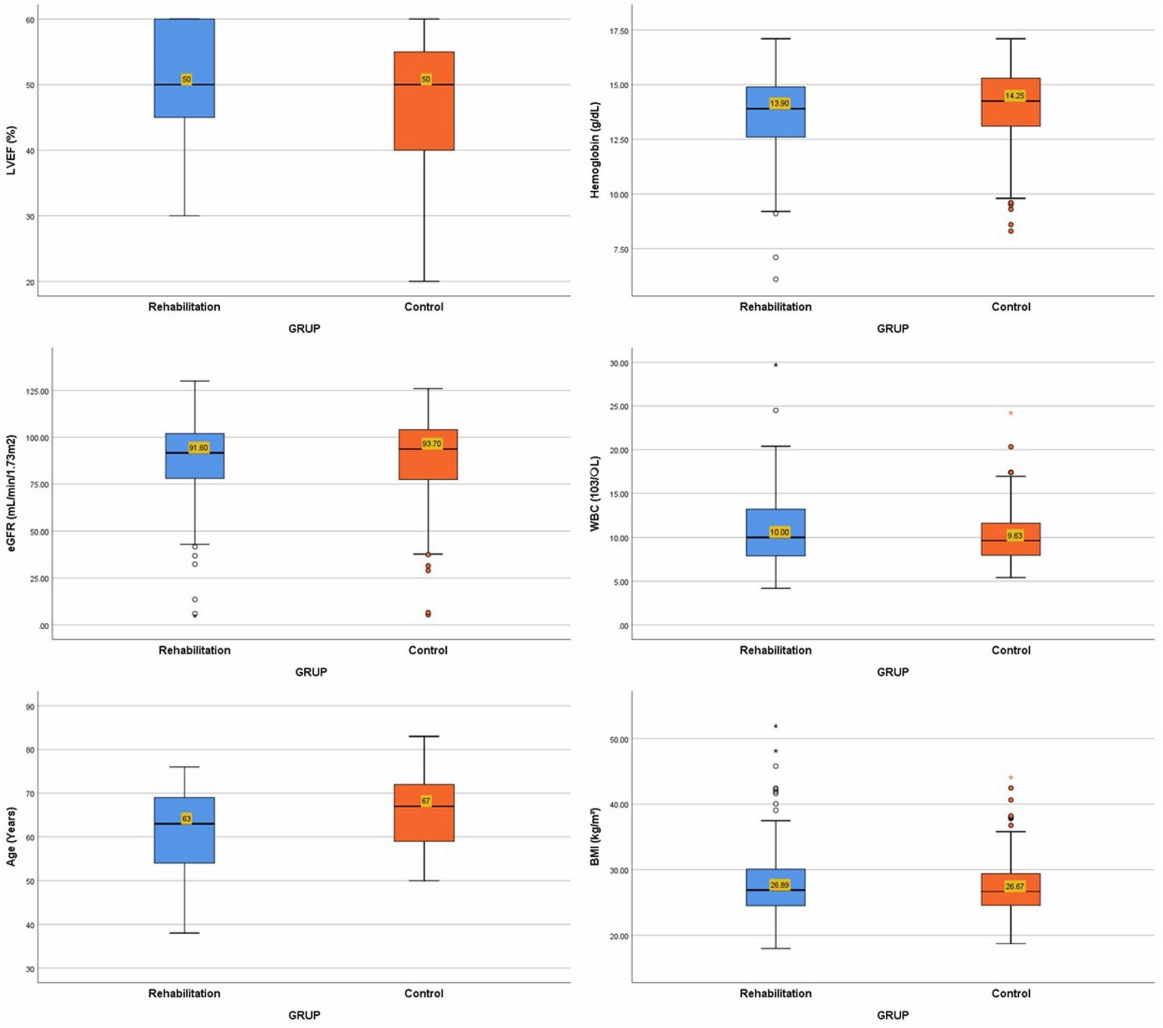
Comparison of the ordinal baseline characteristics of the study patients in the cardiac rehabilitation training group and the control group.

Baseline demographic, clinical, and laboratory characteristics were compared between the two groups. The mean age of all patients was 63.8 years (±9.3), with 55.3% (188 patients) being male. Both age and gender distribution were similar between the groups. The most common comorbidity was hypertension (64.1%), and 48.5% of patients had a prior history of coronary artery disease, with no significant difference in comorbidities between the groups. WBC count, hemoglobin levels, and eGFR were comparable across both groups. The mean left ventricular EF (%EF) of all patients was 48.9% (±9.5), with no significant intergroup differences (Figure 1).

**Figure 2:**
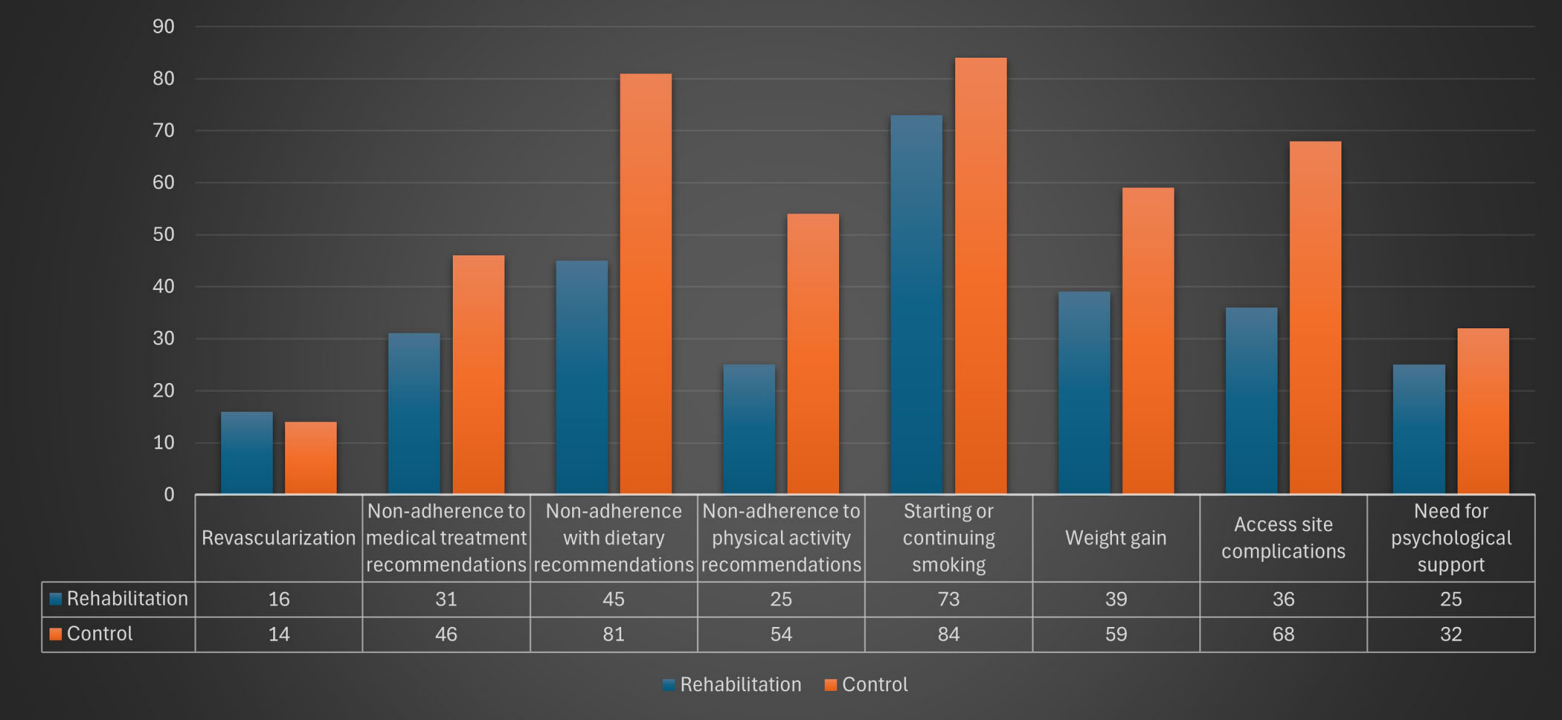
Comparison of acute coronary syndromes in the cardiac rehabilitation training group and the control group.

The most common type of ACS presentation was NSTEMI, observed in 76.2% of the patients (72.9% in the CR training group and 79.4% in the control group), with no significant differences between the groups (Figure 2).

The mean hospital stay was 5 days (interquartile range: 4–7 days) and did not differ significantly between the groups. A detailed comparison of demographic, clinical, and laboratory findings is provided in Table 1.

**Table 1:** Comparison of baseline clinical characteristics of study groups.

Patients characteristics	All patients (*N* = 340)	Cardiac rehabilitation training group (*N* = 170)	Control group (*N* = 170)	Overall *p* value
Age (year)	63.8 ± 9.3	62.4 ± 9.5	65.2 ± 8.3	0.49
Sex (Male)	188 (55.3)	91 (53.5)	97 (57.1)	0.81
BMI (kg/m²)	27.6 ± 5.0	27.9 ± 5.8	27.3 ± 4.4	0.78
Hypertension	218 (64.1)	107 (62.9)	111 (65.3)	0.65
Diabetes mellitus	135 (39.7)	61 (35.9)	74 (43.5)	0.15
Recent smoker	157 (46.2)	74 (43.5)	83 (48.8)	0.33
Hyperlipidemia	37 (10.9)	17 (10.10)	20 (11.8)	0.60
Heart Failure	29 (8.5)	17 (10.0)	12 (7.1)	0.33
Chronic renal disease	49 (14.4)	22 (12.9)	27 (15.9)	0.44
Coronary artery disease	165 (48.5)	90 (52.9)	75 (44.1)	0.10
Anemia	16 (4.7)	7 (4.1)	9 (5.3)	0.61
*Diagnosis at admission*				
STEMI	69 (20.3)	41 (24.1)	28 (16.5)	0.08
NSTEMI	259 (76.2)	124 (72.9)	135 (79.4)	0.16
UAP	12 (3.5)	5 (2.9)	7 (4.1)	0.56
WBC (10^3^/μL)	10.4 ± 3.5	10.6 ± 3.8	10.2 ± 3.2	0.88
eGFR (mL/min/1.73 m^2^)	87.8 ± 22.0	87.1 ± 21.8	88.5 (22.2)	0.97
Hemoglobin (g/dL)	13.8 ± 1.9	13.6 ± 1.9	13.9 ± 1.8	0.91
LVEF (%)	48.9 ± 9.5	50.6 ± 8.9	47.2 ± 9.8	0.69
The duration of hospitalization (day)	5 (4–7)	5 (4–7)	5 (4–6)	0.89

*Note:* Values are mean ± SD, median (IQR) or *n* [*n*/N if missing data] (%). Interquartile range [25% percentile to 75% percentile].

Abbreviations: BMI, body mass index; GFR, glomerular filtration rate; LVEF, left ventricular ejection fraction; NSTEMI, non‐ST elevation myocardial infarction; STEMI, ST‐elevation myocardial infarction; WBC, white blood cell; UAP, unstable angina pectoris.

During the 12‐month follow‐up period, all 340 participants remained under observation, and no deaths were recorded in either group. In the extended follow‐up subset (*n* = 100), adherence to dietary and physical activity recommendations remained significantly higher in the CR group at 18 months (dietary: 68.4% vs. 45.2%, *p* = 0.012; physical activity: 78.9% vs. 60.5%, *p* = 0.021) and 24 months (dietary: 63.2% vs. 42.9%, *p* = 0.028; physical activity: 73.7% vs. 54.8%, *p* = 0.036), though some attenuation of effect size was observed over time. Follow‐up attendance for scheduled clinic visits and telephone interviews was 100%, supported by dedicated coordination and reminder systems. In the intervention group, 158 out of 170 patients (92.9%) successfully completed the entire 12‐week CR program. The remaining 12 participants discontinued rehabilitation prematurely due to personal or logistical reasons but continued with follow‐up assessments and were analyzed according to the intention‐to‐treat principle.

At 1‐year follow‐up, clinical and behavioral outcomes were systematically assessed to evaluate the impact of CR training. Key outcome measures included revascularization rates, adherence to medical and lifestyle recommendations, weight changes, procedural complications, and psychosocial needs.

The CR training group showed significantly better adherence to lifestyle modifications. Non‐adherence to dietary recommendations was significantly lower in this group compared to the control group (26.5% vs. 47.6%, *p* < 0.01). Similarly, non‐adherence to physical activity recommendations was lower in the CR training group (14.7% vs. 31.8%, *p* < 0.01). This suggests a meaningful positive effect of rehabilitation training on lifestyle behavior.

Weight gain was less frequent in the CR training group compared to the control group (22.9% vs. 34.7%, *p* = 0.017). Additionally, access site complications were less common in the CR training group (21.2% vs. 40%, *p* < 0.01). These findings indicate a potential benefit of CR in reducing both lifestyle‐related and procedural adverse outcomes.

**Figure 3:**
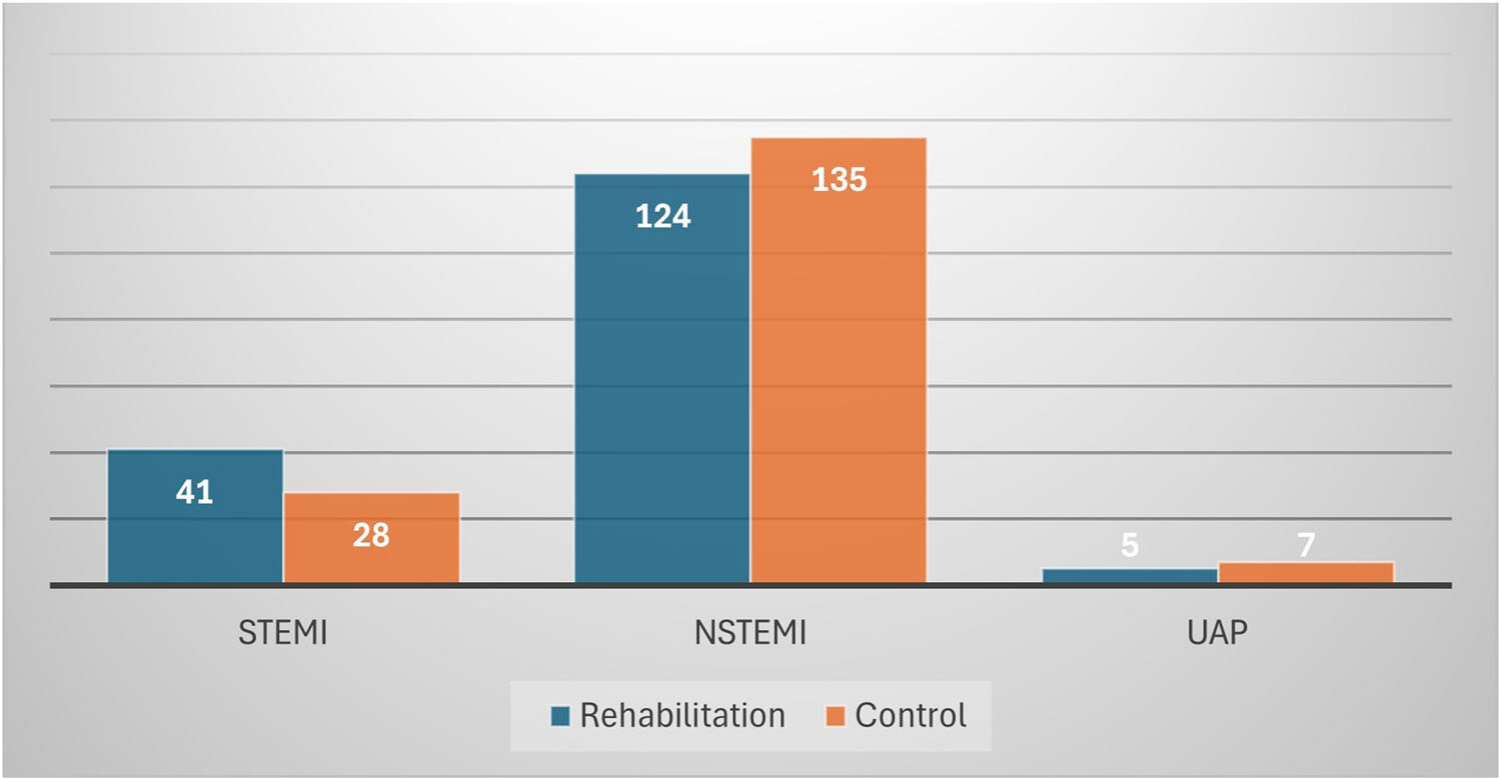
Comparison of study endpoints in the cardiac rehabilitation training group and the control group.

However, there were no significant differences between the groups regarding non‐adherence to medical treatment (*p* = 0.118), revascularization rates (*p* = 0.244), smoking status (*p* = 0.276), or the need for psychological support (*p* = 0.489). Regarding psychological outcomes, baseline PHQ‐9 scores were similar between groups (mean 7.6 ± 5.2 in the CR group vs. 7.9 ± 5.0 in the control group, *p* = 0.58). Similarly, baseline GAD‐7 scores showed no significant difference (mean 6.9 ± 4.4 vs. 7.1 ± 4.3, *p* = 0.66). At 12‐month follow‐up, the CR group showed greater improvement in depression scores (mean change −2.8 ± 3.1 vs. −1.7 ± 2.9, *p* = 0.001). Anxiety scores also improved more substantially in the rehabilitation group (mean change −2.5 ± 2.8 vs. −1.4 ± 2.6, *p* = 0.001). The proportion of patients developing clinically significant worsening in psychological status was lower in the CR group compared to controls (9.4% vs. 17.1%, *p* = 0.033). This suggests that CR may provide psychological benefits beyond lifestyle modification (Table 2, Figure 3).

**Table 2:** Comparison of study outcomes at 1‐year follow‐up between patients receiving cardiac rehabilitation training and those discharged with routine recommendations.

Patients outcomes	All patients (*N* = 340)	Cardiac rehabilitation training group (*N* = 170)	Control group (*N* = 170)	Overall *p* value
Revascularization	30 (8.8)	16 (9.4)	14 (8.2)	0.70
Non‐adherence to medical treatment recommendations	77 (22.6)	31 (18.2)	46 (27.1)	0.052
Non‐adherence with dietary recommendations	126 (37.1)	45 (26.5)	81 (47.6)	<0.01
Non‐adherence to physical activity recommendations	79 (23.2)	25 (14.7)	54 (31.8)	<0.01
Starting or continuing smoking	157 (46.2)	73 (42.9)	84 (49.4)	0.23
Weight gain	98 (28.8)	39 (22.9)	59 (34.7)	0.017
Access site complications	104 (30.6)	36 (21.2)	68 (40)	<0.01
Need for psychological support	57 (16.8)	25 (14.7)	32 (18.8)	0.31
*Psychological outcomes*				
*Depression (PHQ‐9)*				
Baseline score, mean ± SD	7.8 ± 5.1	7.6 ± 5.2	7.9 ± 5.0	0.58
12‐month score, mean ± SD	5.6 ± 4.8	4.8 ± 4.5	6.2 ± 5.0	0.006
Mean change from baseline	−2.2 ± 3.0	−2.8 ± 3.1	−1.7 ± 2.9	0.001
Moderate‐severe symptoms (PHQ‐9 ≥ 10) at 12 months	51 (15.0)	18 (10.6)	33 (19.4)	0.021
*Anxiety (GAD‐7)*				
Baseline score, mean ± SD	7.0 ± 4.3	6.9 ± 4.4	7.1 ± 4.3	0.66
12‐month score, mean ± SD	5.1 ± 4.2	4.4 ± 3.9	5.7 ± 4.4	0.003
Mean change from baseline	−1.9 ± 2.7	−2.5 ± 2.8	−1.4 ± 2.6	0.001
Moderate‐severe symptoms (GAD‐7 ≥ 8) at 12 months	58 (17.1)	21 (12.4)	37 (21.8)	0.019
Clinically significant worsening in psychological status^a^	45 (13.2)	16 (9.4)	29 (17.1)	0.033

*Note:* Values are presented as *n* (%) unless otherwise specified.

^a^Defined as increase of ≥5‐points in either PHQ‐9 or GAD‐7 from baseline to 12 months, or development of moderate‐to‐severe symptoms in previously asymptomatic patients.

Exercise‐related adverse events occurred in 17 patients (10%) in the CR group. These included transient exercise‐induced chest discomfort without ECG changes in eight patients (4.7%), exercise‐induced ventricular ectopy not requiring intervention in five patients (2.9%), excessive blood pressure response in three patients (1.8%), and minor musculoskeletal injuries in four patients (2.4%). All events resolved with temporary modification or discontinuation of exercise, and no patient withdrew from the study due to adverse events. No serious cardiac complications (arrhythmias requiring intervention, myocardial infarction, cardiac arrest) occurred during exercise sessions.

Regarding dietary intervention‐related adverse events, mild gastrointestinal symptoms (primarily bloating and changes in bowel habits) were reported by 12 patients (7.1%) in the rehabilitation group during the initial weeks of dietary modification, all of which resolved with minor adjustments to the dietary plan. No significant adverse nutritional outcomes were observed.

There was no significant difference in MACE between groups during the 12‐month follow‐up period (CR: six patients [3.5%] vs. control: nine patients [5.3%], *p* = 0.42).

A Cox regression analysis was performed to identify factors influencing revascularization within the first year. Non‐adherence to medical treatment and dietary recommendations were significantly associated with 1‐year revascularization in both univariate and multivariate analyses. In the multivariate analysis, non‐adherence to medical treatment was associated with a HR of 0.325 (95% CI: 0.109–0.758, *p* = 0.019), and non‐adherence to dietary recommendations was associated with an HR of 0.347 (95% CI: 0.133–0.910, *p* = 0.031).

After adjustment for socioeconomic factors, baseline medication use, and psychosocial variables, these associations remained significant. Among the additional covariates, lower education level (HR 1.89, 95% CI: 1.14−3.12, *p* = 0.013) and baseline depression scores (PHQ‐9 ≥ 10) (HR 1.76, 95% CI: 1.09−2.84, *p* = 0.021) were independently associated with increased risk of revascularization. Interestingly, pre‐admission statin use was associated with lower revascularization risk (HR 0.68, 95% CI: 0.47−0.98, *p* = 0.042), suggesting potential protective effects of previous lipid‐lowering therapy. Other socioeconomic and medication variables did not demonstrate statistically significant associations with the primary outcome.

These results suggest that behavioral factors, particularly adherence to medical and dietary recommendations, significantly impact the need for subsequent revascularization. Despite no significant difference in overall revascularization rates between the two groups, the improved adherence patterns in the rehabilitation group may translate into long‐term clinical benefits.

No significant associations were found between other endpoints and revascularization (Table 3).

**Table 3:** Univariate and multivariate Cox regression analysis for predictors of revascularization at 1‐year follow‐up.

Patients outcomes	Univariate analysis			Multivariate analysis		
	HR	95% CI	*p*	HR	95% CI	*p*
Non‐adherence to medical treatment recommendations	0.297	0.099−0.693	0.014	2.840	1.320−6.110	0.007
Non‐adherence with dietary recommendations	0.340	0.130−0.887	0.028	2.420	1.080−5.410	0.032
Non‐adherence to physical activity recommendations	0.826	0.338−2.021	0.675	0.870	0.353−2.190	0.756
Starting or continuing smoking	1.020	0.498−2.090	0.957	0.985	0.477−2.034	0.969
Weight gain	1.235	0.578−2.638	0.586	1.354	0.617−2.973	0.451
Access site complications	0.825	0.367−1.853	0.642	0.851	0.377−1.920	0.703
Need for psychological support	1.551	0.648−3.521	0.339	1.498	0.622−3.605	0.370
*Socioeconomic factors*						
Lower education level (≤high school)	1.76	1.08−2.87	0.024	1.89	1.14−3.12	0.013
Lower income bracket (below median)	1.45	0.92−2.28	0.110	1.37	0.85−2.19	0.194
Unemployment	1.38	0.89−2.14	0.152	1.29	0.82−2.04	0.273
*Baseline medication use*						
Pre‐admission antiplatelet therapy	0.82	0.56−1.21	0.317	0.91	0.61−1.36	0.648
Pre‐admission statin therapy	0.72	0.51−1.03	0.068	0.68	0.47−0.98	0.042
Pre‐admission beta‐blocker therapy	0.88	0.60−1.29	0.511	0.93	0.62−1.38	0.704
Pre‐admission ACE‐I/ARB therapy	0.91	0.64−1.30	0.612	0.95	0.65−1.39	0.788
*Psychosocial variables*						
Baseline depression (PHQ‐9 ≥ 10)	1.69	1.07−2.69	0.026	1.76	1.09−2.84	0.021
Baseline anxiety (GAD‐7 ≥ 8)	1.44	0.93−2.22	0.101	1.21	0.76−1.92	0.417

Abbreviations: ACE‐I, angiotensin‐converting enzyme inhibitor; ARB, angiotensin receptor blocker; CI, confidence interval; GAD‐7, Generalized Anxiety Disorder‐7; HR, hazard ratio; PHQ‐9, Patient Health Questionnaire‐9.

## 4 Discussion

Our randomized controlled trial provides robust evidence that structured CR significantly improves multiple dimensions of post‐ACS care. Three key findings emerge from this study [1]: CR produces superior adherence to lifestyle modifications compared to standard care alone [2], these behavioral changes translate into tangible clinical benefits including reduced weight gain and procedural complications, and [3] medication and dietary non‐adherence remain potent predictors of revascularization risk. These results align with but substantially extend previous literature by demonstrating comprehensive benefits across both behavioral and hard clinical endpoints.

The observed improvements in dietary (73.5% vs. 52.4%) and physical activity adherence (85.3% vs. 68.2%) in the CR group merit careful consideration. While these findings corroborate previous reports of CR's effectiveness in promoting lifestyle changes [15, 16], our study uniquely demonstrates that these benefits persist beyond the active intervention period in a substantial proportion of patients. However, the 20%−25% non‐adherence rate in the CR group suggests room for improvement. This residual non‐adherence may reflect known barriers including socioeconomic factors [17] or psychological comorbidities [18], which were not fully addressed in our intervention. Future CR programs might benefit from incorporating more intensive behavioral support tailored to high‐risk subgroups.

Our findings regarding weight management (22.9% vs. 34.7% weight gain) contribute to ongoing debates about optimal approaches to cardiovascular risk reduction. While the observed effect size is modest compared to intensive weight loss programs [19], the sustainability of our results at 12 months compares favorably with previous reports of CR effects [20]. Notably, the reduced access site complications (21.2% vs. 40%) represent a novel finding that may reflect CR's multimodal benefits on vascular health through improved physical conditioning and inflammatory profiles [21]. This unexpected benefit warrants further mechanistic investigation.

After correcting for variable coding, the study confirmed non‐adherence to medical treatment and dietary recommendations as significant independent predictors of increased revascularization risk. This finding highlights the critical role of adherence in preventing disease progression and repeat interventions. Non‐adherence to prescribed therapies has been consistently associated with poorer clinical outcomes, including increased rates of mortality and rehospitalization [22, 23, 24, 25]. Efforts to improve adherence should remain a priority in post‐ACS care, with CR programs serving as a valuable tool for reinforcing the importance of compliance.

Despite the demonstrated benefits of CR, participation rates remain suboptimal in real‐world settings [10, 26]. Barriers such as lack of referral, logistical constraints, and patient reluctance often limit enrollment. In this study, the randomized design ensured equal access to rehabilitation services, allowing for an unbiased evaluation of their impact. However, broader implementation strategies are needed to enhance program uptake, particularly among underserved populations.

The findings of this study are consistent with prior evidence demonstrating the efficacy of CR in improving functional capacity, quality of life, and adherence behaviors. However, this study adds to the existing literature by specifically examining the effects of rehabilitation on dietary adherence, physical activity, and complications such as weight gain and access site issues. Additionally, the use of Cox regression analysis to identify predictors of revascularization provides valuable insights into the factors influencing long‐term outcomes, which has been less explored in previous research.

This study underscores the need for integrating CR programs into routine post‐ACS care to enhance patient compliance and improve outcomes. Healthcare providers should prioritize the referral of eligible patients to such programs and address barriers to participation. Policymakers and healthcare institutions should consider implementing initiatives to expand access to rehabilitation services, particularly in regions with limited availability.

While our extended follow‐up subset analysis suggests that behavioral changes are partially sustained up to 24 months, the observed attenuation in adherence rates underscores the need for longer‐term studies. Further research is warranted to evaluate the durability of CR benefits beyond 2 years, including mortality, recurrent ACS, and quality of life, as well as strategies to reinforce adherence. Studies exploring strategies to enhance program adherence, such as telehealth‐based rehabilitation or mobile health interventions, may also provide valuable insights. Additionally, investigating the effects of rehabilitation across diverse patient populations and healthcare settings will help tailor interventions to meet the needs of different communities.

## 5 Limitations

While our study provides compelling evidence for CR benefits, several limitations contextualize the findings [1]: The single‐center design, though ensuring protocol consistency, may limit generalizability to diverse healthcare settings—a concern echoed in recent systematic reviews [2, 27]. Our adherence measures, while multimodal, still relied partly on self‐report, potentially overestimating true adherence rates as demonstrated in validation studies [3, 28]. The exclusion of high‐risk patients (e.g., advanced heart failure) leaves unanswered questions about CR's effectiveness in these vulnerable populations where benefits might be magnified. These limitations highlight critical directions for future research.

## 6 Conclusion

This study demonstrates that CR training significantly improves adherence to dietary and physical activity recommendations and reduces the incidence of weight gain and access site complications in patients following ACS. These findings highlight the potential benefits of structured rehabilitation programs in enhancing post‐ACS care and promoting better health outcomes.

Furthermore, non‐adherence to medical treatment and dietary recommendations were independently associated with an increased risk of revascularization within 1 year. These results underscore the critical importance of adherence to prescribed therapeutic and lifestyle modifications in minimizing the need for repeat interventions and improving long‐term cardiovascular health.

Incorporating CR programs into routine post‐ACS care may serve as a valuable strategy to enhance patient compliance, reduce complications, and improve overall outcomes. Further research, including larger studies with extended follow‐up periods, is warranted to explore the broader impact of such interventions across diverse patient populations and to identify strategies for sustaining adherence beyond the initial post‐ACS year.

## Author Contributions

F.P. and H.T. were the primary contributors, as they conceived the initial idea for the study and played a central role in developing the theoretical framework and performing the computations. Both also contributed to verifying the analytical methods and participated in discussions on the results and the final manuscript. E.Ö. and E.A. collaborated on developing the theory and performing computations, while also contributing to the discussions and final manuscript. A.N.K. and F.C. focused on verifying the analytical methods and participated in result discussions and manuscript preparation. H.K. and Ç.B.Ç. were involved in supervising the findings and contributed to discussions on the results and the final manuscript. All authors collectively engaged in reviewing and refining the final manuscript, ensuring a collaborative effort in completing the research.

## Ethics Statement

Ethical approval for the research described in the study was sought and granted by the Van Education and Research Hospital Clinical Research Ethics Committee. The approval was issued on 29.07.2021, with the decision numbered 2021/15.

## Consent

All participants involved in this study provided informed consent before their inclusion. The consent process was conducted according to ethical standards, explaining the study's purpose, procedures, potential risks, and benefits. Participants were assured of confidentiality, voluntary participation, and the right to withdraw without consequences. Participants in this study were informed that the data collected may be used for publication purposes while ensuring anonymity and confidentiality. The consent for publication includes an understanding that no personally identifiable information will be disclosed.

## Data Availability

Data supporting the findings of this study are available from the corresponding author, upon reasonable request, via the e‐mail address drfuatpolat@gmail.com. The analysis and findings rely on standard statistical methods and commercially available software tools. For any inquiries related to the methodology or data analysis, please contact the corresponding author.
